# Perspectives of bilateral thoracic sympathectomy for treatment of heart failure

**DOI:** 10.6061/clinics/2021/e3248

**Published:** 2021-07-26

**Authors:** Raphael dos Santos Coutinho e Silva, Fernando Luiz Zanoni, Rafael Simas, Luiz Felipe Pinho Moreira

**Affiliations:** I Laboratorio Cirurgico de Pesquisa Cardiovascular (LIM-11), Instituto do Coracao (Incor), Hospital das Clinicas HCFMUSP, Faculdade de Medicina, Universidade de Sao Paulo, Sao Paulo, SP, BR.; II The Rolf Luft Research Center for Diabetes and Endocrinology, Karolinska Institutet, Stockholm, Sweden.

**Keywords:** Heart Failure, Sympathectomy, Myocardial Infarction, Dilated Cardiomyopathy, Pulmonary Hypertension

## Abstract

Surgical neuromodulation therapies are still considered a last resort when standard therapies have failed for patients with progressive heart failure (HF). Although a number of experimental studies have provided robust evidence of its effectiveness, the lack of strong clinical evidence discourages practitioners. Thoracic unilateral sympathectomy has been extensively studied and has failed to show significant clinical improvement in HF patients. Most recently, bilateral sympathectomy effect was associated with a high degree of success in HF models, opening the perspective to be investigated in randomized controlled clinical trials. In addition, a series of clinical trials showed that bilateral sympathectomy was associated with a decreased risk of sudden death, which is an important outcome in patients with HF. These aspects indicates that bilateral sympathectomy could be an important alternative in the treatment of HF wherein pharmacological treatment barely reaches the target dose.

## BACKGROUND

Heart failure (HF) affects approximately 2% of the adult population in developed countries. This number increases to 10% among people over the age of 70 years (1). Even in developed countries, 50% of patients with HF die within 5 years after diagnosis. HF progression is highly linked to an overactivated sympathetic nervous system (SNS) and renin angiotensin aldosterone system (RAAS), which makes them the main targets for medical treatment (2,3). Although current medical treatments have improved survival after diagnosis, mortality remains high (1).

HF patients have been treated with a combination of angiotensin-converting enzyme inhibitor (ACEI) or angiotensin-II receptor blocker (ARB) if ACEI is not tolerated, a β-blocker (BB), and a mineralocorticoid receptor antagonist (MRA) (4). Most recently, a new drug was approved for treatment of HF, a first-class angiotensin receptor neprilysin inhibitor (ARNI) that contains an ARB, sacubitril, and an inhibitor of neprilysin, valsartan (5,6).

Even though clinical treatment guidelines support the combined therapy based on large randomized controlled trials, medication use and dosage in clinical practice are suboptimal. In the United States, the CHAMP-HF registry presented significant gaps in the use and dose of the current medical treatment for HF. Among patients eligible for the combined therapy, 26.6%, 33%, and 76.6% did not receive ACEI/ARB/ARNI, BB, and MRA, respectively. Additionally, less than 30% of the patients received the target doses of BB and ACEI/ARB/ARNI. Only 1% of the patients received optimal treatment (ACEI/ARB/ARNI+BB+MRA) and target dosage (7).

Due to the continuing high mortality in patients with HF and the continuous progression despite treatment, strategies to improve this setting continues to be required. Blockade through thoracic sympathectomy might be an alternative for halting disease progression using different pathways.

## PATHOPHYSIOLOGY

The most recent classification of HF stratifies into 2 groups based on the contractile function of the left ventricle. Hence, HF is classified in HF with reduced ejection fraction (HFrEF), i.e., patients with left ventricular ejection fraction (LVEF) lower than 40%, and HF with preserved ejection fraction (HFpEF), i.e., patients with LVEF higher than 50%. A more recent classification, HF with midrange ejection fraction (HFmrEF) englobes those with LVEF between 41 to 49%. On this review, we will be focusing on HFrEF, once the development of this HF is directly linked to compensatory mechanisms that aim to improve cardiac function. HFrEF is mainly caused by ischemic heart disease, dilated cardiomyopathies of different etiologies or right heart failure secondary to pulmonary arterial hypertension. As for the main compensatory mechanisms, there are the neurohormonal systems (SNS and RAAS), Frank Starling mechanism (stretch-induced increase of preload) and myocardial structural changes (hypertrophy and hyperplasia) (2).

Regardless of the cause of HF, a reduction in cardiac output leads to an increase in the contraction force by elongation of the cardiomyocyte sarcolemma (8). In addition, the SNS and RAAS are activated and have pivotal roles in HFrEF progression. The release of catecholamines by the SNS, mainly norepinephrine, enhances heart rate and myocardial contractility, which in the early clinical phase of the disease, preserves cardiac output (9-12). Additionally, circulating norepinephrine activates the RAAS, with subsequent release of angiotensin-II. Angiotensin-II enhances the effects of norepinephrine, with systemic vasoconstriction, as well as water and sodium retention, augmenting the venous return to the heart and increasing cardiac filling pressure (13,14).

When healthy, the heart is able to derive energy from fatty acids, glucose, ketone bodies, and amino acids. Under normal conditions, fatty acids are the main source of energy and are responsible for up to 90% of ATP production. These metabolic flexibilities support increased cardiac workload (15). Another important compensatory mechanism is myocardial remodeling through cardiomyocyte hypertrophy and an accelerated apoptosis/regeneration cycle (2,16).

However, constant activation of the aforementioned mechanisms leads to decompensation. Continued increases in chronotropy and inotropy reduce coronary perfusion, leading to myocardial ischemia and downregulation of myocardial beta-adrenergic receptors, especially beta-1 receptor, thus reducing inotropic response (17). Furthermore, systemic vasoconstriction elevates afterload and augments ventricular wall stress (18). Consequently, there is an increase in myocardial oxygen demand despite the limited supply due to myocardial ischemia (19).

As HFrEF progresses, the main source of energy shifts from fatty acids to glucose oxidation. The diminished oxygen supply reduces cardiomyocyte production of high-energy phosphate availability, which is responsible for transporting ATP to the myofibers. This metabolism changes with the accumulation of toxic intermediates, leads to mitochondrial dysfunction and increased oxidative stress (15). Damage by reactive oxygen species (ROS) leads to further impairment of cardiac energy homeostasis due to poor mitochondrial repair capacity (20). As energy depletes and excessive elongation of the fiber leads to the failure of muscle contractile unity, apoptosis and necrosis intensify. Cardiomyocytes are replaced by fibrotic tissue, and the ventricular remodeling process becomes pathological (21).

The development of myocardial fibrosis alters the mechanical properties of the heart during contraction by untying the cardiomyocyte contact and limiting the oxygen supply. The band separation of the cardiomyocytes leads to a non-uniform anisotropy in conduction speed, inducing micro-ischemic conditions and prolonging the duration of action potential. Increased SNS activity also has an arrhythmogenic effect on HFrEF. The constant activation of B1 receptors induces refractory tachycardia and malignant ventricular arrhythmias. In addition, fibrotic tissue can act as a potential trigger for reentry arrhythmia and is associated with sudden death, the second most prevalent outcome in patients with HFrEF (22,2).

Along with life-threatening arrhythmias, HFrEF progression can lead to the development of pulmonary hypertension (PH). An elevation in the left ventricular (LV) filling pressure causes an increase in pulmonary venous pressure (23,24). The resulting vascular remodeling increases pulmonary vascular resistance, leading to a high right ventricular (RV) afterload, further deteriorating the damaged heart. PH can also cause HFrEF as idiopathic PH leads to increased RV afterload due to pulmonary vascular remodeling (25,26). In addition, the SNS promotes pulmonary vascular remodeling and is further activated by it (27,28). Regardless of whether PH is a cause or consequence of HFrEF, the presence of PH is associated with a poor prognosis for mortality in patients with HFrEF (29).

## PHARMACOLOGICAL THERAPY

With increasing stiffness of the heart, cardiac systolic function decreases even further. Eventually, the compensatory mechanisms are overwhelmed and unable to sustain cardiac function without further decompensation. The neurohormonal system is the main target for the treatment of HFrEF to dampen this vicious cycle (9-12).

The ACEI/ARB/ARNI and MRA target the RAAS, while the SNS is directly targeted by the BB (5-7). Antiarrhythmic drugs such as amiodarone and implantable cardioverter defibrillators (ICDs) are also important in preventing sudden death from life-threatening arrhythmias (30,31). However, as previously mentioned, less than 30% of patients on gold standard therapy receive the optimal dosage (7). Since pharmacological therapy has a low percentage of optimal dosage, surgical neuromodulation therapies could offer a greater benefit, particularly those that target the SNS.

## NEUROMODULATION THERAPIES

Renal denervation (RD) has been studied using various experimental models and clinical trials. RD is based on dampening renal sympathetic activity, which is responsible for increasing sodium and fluid retention and activating the RAAS. In experimental models of myocardial infarction in rats, RD was associated with less fibrosis and cardiac remodeling, better cardiac function, decreased SNS activation, and improved hemodynamics (32,33). In the clinical setting, although the safety of the RD procedure is assured, its efficacy in patients with HFrEF is not as clear. The REACH trial showed that RD was associated with symptom and exercise capacity improvements; however, the small group size and lack of control groups preclude any robust conclusions (34).

Another important point to discuss is whether reinnervation occurs after RD. Using a sheep model, Booth et al. showed that 5 months after the RD procedure, almost complete functional and anatomical reinnervation occurred. Eleven months later, no differences were found in RD and non-RD animals, in relation to the renal distribution of afferent and efferent nerves and renal NE levels (35). There is still a lack of clinical trials with long-term follow-up and strong clinical endpoints to demonstrate whether RD might be an effective alternative for patients with HFrEF.

Vagal nerve stimulation (VNS) is another therapy option, which is based on the aforementioned disbalance between the SNS and the parasympathetic nervous system in HFrEF. Rather than attempting on or dampen SNS activation, VNS tries to balance the autonomic nervous system by stimulating the vagal nerve with an implanted device. In experimental studies using rat models of HFrEF, VNS baroreflex activation decreased cardiac oxygen consumption, inflammation, SNS activity, and overall mortality (36-38). In a canine model of HFrEF, VNS was associated with improved LV function and decreased levels of several cardiac biomarkers (39,40).

The success of VNS in experimental models has not been replicated in clinical trials. In the randomized, sham-controlled, double-blind NECTAR-HF trial, VNS did not improve LV remodeling for 6 months, nor did it achieve any of the secondary efficacy endpoints (41). INOVATE-HF was another study that investigated VNS use in HFrEF. This was a multicenter, randomized trial with chronic HFrEF patients (NYHA III, left ventricular ejection fraction <40%) who were enrolled for 16 months. The trial concluded that VNS does not reduce mortality in patients with chronic HFrEF or diminish HF events (42).

Pulmonary artery denervation (PADN) is an alternative option for primary pulmonary arterial hypertension (PAH) in an attempt to prevent HFrEF development. The PADN mechanism is based on the removal of sympathetic nerves from the main pulmonary artery trunk, thereby inhibiting excessive activation of the SNS. An experimental model of PH showed improved hemodynamics and diminished pulmonary artery remodeling (43). In patients with idiopathic PH, PADN decreased the mean pulmonary arterial pressure and improved the 6-minute walk test in a 3-month follow-up (44). However, the small number of patients and the lack of larger studies preclude the assessment of PADN efficacy.

## THORACIC SYMPATHECTOMY

Thoracic sympathectomy was first described as an alternative for arrhythmia control in patients with angina and ventricular arrhythmia (45). Thoracic sympathectomy surgery has improved and is now performed by thoracoscopy with high levels of success and minimal collateral effects. One of the main sympathectomy mechanisms is the increase in the fibrillation threshold, in addition to enhancing efferent vagal nerve activity. Besides to ICD therapy, left thoracic sympathectomy (LS) is one of the main treatment options for patients with long QT syndrome, sustained ventricular tachycardia, and other heart rhythm disorders (46).

Although LS has positive results in controlling arrhythmias, nervous plasticity of the stellate ganglion was observed after LS treatment. Unilateral sympathectomy causes hypertrophy of the contralateral ganglion (47,48). This can be seen when LS is compared with bilateral sympathectomy (BS). Furthermore, LS and BS were compared in a rat model of myocardial infarction, and BS was shown to be effective in protecting LV function and morphology, while LS failed to do so (49). In patients with ventricular arrhythmia, BS was more beneficial than LS in terms of arrhythmia control and decreased ICD shocks (47). In developing countries, this is an important result, considering the high cost of such mechanical devices. In comparison, BS surgery is a safe and low-cost procedure.

One of the main concerns about performing BS rather than unilateral sympathectomy was the maintenance of a minimal adrenergic tonus in a patient with HFrEF. A clinical trial verified the safety of BS in patients with ventricular tachyarrhythmia (47). Another study confirmed the safety of this procedure in patients with severe HFrEF. Also, it showed that epidural thoracic blockade was responsible for completely decreasing the sympathetic influence (50). In an experimental setting, a study evaluated the effects of BS on physiological scenarios. Interestingly, this study suggested the possibility of an extracardiac sympathetic compensation pathway that sustains the sympathetic tonus. The higher concentration of peripheral catecholamines and increased heart rate at rest in BS rats compared to non-operated rats supported this hypothesis (51).

The potential benefits of LS were assessed in 10 patients with dilated cardiomyopathy (DCM) and NYHA II or III with reduced LVEF. On a short follow-up of 6 months, LS improved the LVEF, exercise performance, and quality of life. These results showed that LS is feasible; however, a larger study is required to assess its long-term effects (52,53).

Recently, BS was performed in a patient with nonischemic DCM, NYHA class IV, and 15% LVEF. After 1 year, the patient had no ICD shock, improved LVEF to 25%, and was removed from the transplant list, showing the potential benefit of BS in a HFrEF patient (54). In another study, an epidural thoracic blockade was performed in 20 patients, followed for 30 days, with an increase in LVEF, reduction in LV dilatation, and improvement in NYHA class. Although it was a temporary blockade, it showed encouraging results (50). However, no other clinical trial determining BS potential benefits for patients with HFrEF has been performed or is currently active.

Although clinical trials are lacking, experimental studies in different DCM models are being published. Zanoni compared the effect of LS to BS as a treatment for myocardial infarction in rats. BS effectively controlled the overactive SNS, preventing LV remodeling and decay of function. In contrast, LS-treated rats showed a loss of wall thickness, increased fibrosis, and decreased heart function. The potential mechanism by which BS acts on ventricular remodeling is by decreasing myocardial apoptosis. BS-treated rats had diminished expression of apoptosis proteins, as LS and untreated rats had increased expression of these proteins. In addition, BS decreased the expression of matrix metalloproteinases, which are known to play a role in myocardial remodeling (49).

Once BS was shown to be superior to LS, BS was tested in a DCM model induced by doxorubicin (55). DCM rats were treated with either BS or conventional ACEI therapy. After dobutamine stimulation, both treatments preserved LV function; however, only BS was able to preserve LVEF and myocardial efficiency under steady-state conditions. Additionally, only BS animals were able to respond adequately to the preload maneuver, with increased preload recruitable stroke work. These different responses in LV function were correlated with histological analyses. On one hand, both treatments decreased LV dilatation; on the other hand, only BS was able to prevent the decrease in LV wall thickness. This prevention was associated with diminished expression of apoptosis proteins after BS treatment and could explain the difference in function from ACEI treatment (55).

In another unpublished experimental study using a PAH model, BS effects were evaluated in both lung microcirculation and in the RV (Unpublished data). BS was successful in preventing lung arterial wall hypertrophy, which is the primary pathological alteration observed in PAH. An interesting finding was the diminished expression of α-smooth muscle actin (α-SMA) by BS, since the augmented expression of this particular protein is associated with endothelial dysfunction and vascular remodeling. BS protected the RV from dilation and hypertrophy by decreasing pulmonary vascular resistance. Consequently, RV function was preserved under steady-state conditions and responsive to preload changes. In untreated PAH, cardiomyocyte hypertrophy was associated with mitochondrial stress, decreased mitochondrial copy number, and increased oxidative stress. BS could decrease mitochondrial stress by enhancing radical scavenging activity and mitigating oxidative stress. These results could indicate a new mechanism by which BS halts the vicious cycle of SNS-induced lesions by protecting the heart from further damage and might contribute to reverse remodeling (Unpublished data).

In both the myocardial infarction and PAH models, the main cause of cardiac dysfunction was derived from an overactivated SNS in response to a lesion. In contrast, the doxorubicin model caused direct injury to cardiomyocytes. This direct injury leads to cardiomyocyte death and replacement of fibrotic tissue. It is important to highlight the success of BS in all three models, even in a direct lesion model. These results support the hypothesis that BS has a protective role in myocardial apoptosis through an unknown pathway. This mechanism might be key in preventing LV remodeling and halting the progression of HFrEF. The doxorubicin model showed that this mechanism might be different from that of ACEI and could be synergistic.

## FUTURE PERSPECTIVES

**Figure 1:**
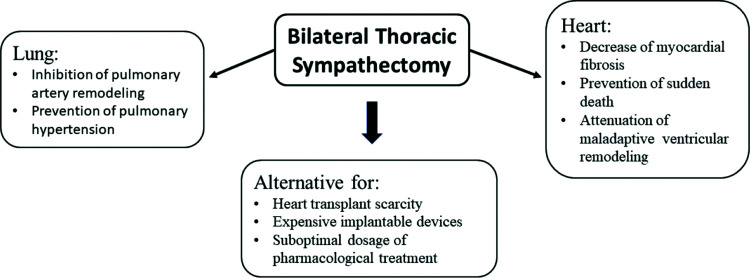
Bilateral sympathectomy potential benefits for patients with heart failure.

Thoracic BS is a minimally invasive and promising procedure for patients with HFrEF (50,54). Experimental (49) and clinical (47,48) studies have shown that BS is superior to LS. Furthermore, BS seems to have a number of potential benefits for patients with HFrEF (Figure 1), such as attenuation of maladaptive ventricular remodeling or even induction of reverse remodeling; prevention of sudden death by decreasing myocardial fibrosis, which is the trigger for life-threatening arrhythmias; inhibition of pulmonary artery remodeling; and consequent prevention of primary or secondary pulmonary hypertension.

The current pharmacological treatment does not prevent the progression of HFrEF, as its dosage is mostly suboptimal in patients (7). In addition, implantable devices, such as LV mechanical assist devices and ICDs are expensive, especially in developing countries. Therefore, along with its potential benefits, BS could be an alternative for the scarcity of heart transplants, the only option for end-stage patients with HFrEF. The mechanisms by which BS acts has already been studied in a number of experimental studies, but with few clinical information, precluding definitive conclusions about its potential benefits. The absence of severe complications and side effects in patients submitted to BS for treatment of ventricular arrhythmias (47) or hyperhidrosis (56) also does not exclude possible risks in HF patients. Randomized controlled trial in patients with HFrEF must be performed to fully assess the effects of thoracic BS and provide real evidence of its perspective as an alternative to the current treatments.

## AUTHOR CONTRIBUTIONS

All the authors were responsible for the manuscript drafting, editing and review.
